# Supplementary material for: The interactome of intact mitochondria by cross-linking mass spectrometry provides evidence for coexisting respiratory supercomplexes
Source: Mol Cell Proteomics. 2017 Dec 8;17(2):216–32. doi: 10.1074/mcp.RA117.000470 (PMC5795388; doi:10.1074/mcp.RA117.000470)
Supplement: Supplemental Data [file supp_RA117.000470_133922_0_supp_23978_fzffwf.zip › spectra_annotation/mito_DR_spectra_annotation/61-1-19-1-19-1.pdf]

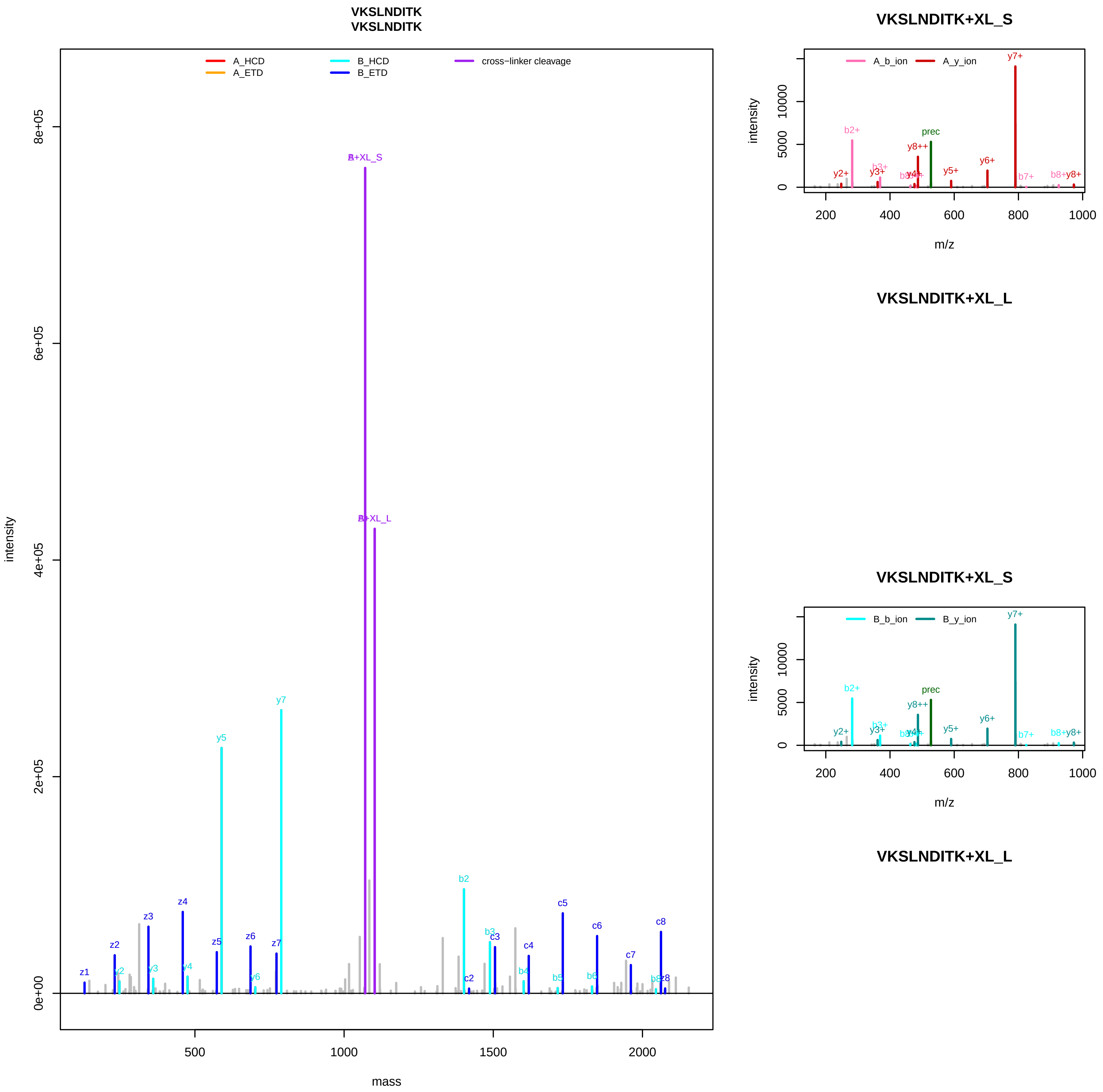
VKSLNDITK
VKSLNDITK
A_HCD
A_ETD
B_HCD
B_ETD
cross−linker cleavage
B+XL_S
B+XL_L
intensity
mass
z1
z2
y2
z3
y3
z4
y4
z5
y5
z6
y6
z7
y7
b2
c2
b3
c3
b4
c4
b5
c5
b6
c6
c7
b8
c8
z8
VKSLNDITK+XL_S
A_b_ion
A_y_ion
y7+
b2+
prec
y8++
y6+
y2+
b3+
y3+
y4+
y5+
b7+
b8+
y8+
m/z
VKSLNDITK+XL_L
VKSLNDITK+XL_S
B_b_ion
B_y_ion
VKSLNDITK+XL_L
